# Potential of circulating tumor DNA to refine immunotherapy

**DOI:** 10.1002/cncr.34714

**Published:** 2023-03-04

**Authors:** Rebecca J. Lee, Jason J. Luke

**Affiliations:** 1 University of Manchester, Manchester, UK; 2 The Francis Crick Institute, London, UK; 3 The Christie NHS Foundation Trust, Manchester, UK; 4 UPMC Hillman Cancer Center, Pittsburgh, Pennsylvania, USA; 5 Department of Medicine, University of Pittsburgh, Pittsburgh, Pennsylvania, USA

In this issue of *Cancer*, Ergolu and colleagues^1^ describe the emerging utility of circulating tumor DNA (ctDNA) as a predictor of immunotherapy treatment outcomes in melanoma.^1^ Although this study is only an initial glimpse into the potential of ctDNA to impact on clinical practice, it seems likely that noninvasive disease assessment will have a major role in oncology moving into the future. Many studies across tumor types have now demonstrated ctDNA as a marker of tumor burden and cancer proliferation.^2,3^ In addition, ctDNA has been shown to be predictive of treatment response across a variety of treatment modalities.^4–7^ In adjuvant bladder cancer, reanalysis of an originally reported negative phase 3 trial suggests that ctDNA is highly predictive of benefit for atezolizumab versus observation. Similarly in colorectal cancer, noninferiority of a ctDNA-guided approach compared to adjuvant chemotherapy has been demonstrated in stage II disease.^8,9^ Eroglu et al.^1^ add to this literature by providing further insight into ctDNA dynamics during immune-checkpoint inhibitors (ICI) in both adjuvant and advanced melanoma. They found that increasing ctDNA levels during ICI was predictive of distant metastatic relapse in the adjuvant setting as well as inferior progression-free survival (PFS) in treatment-naive metastatic disease.

A wide range of ctDNA assays are now in clinical use, ranging from tumor-informed approaches that apply identified mutations into small targeted panels or droplet digital assays, to tumor agnostic approaches that sequence the whole genome or methylome. Which of these approaches is optimal is likely application- or investigation-specific and there are few head-to-head comparisons. One hypothesis would be that given the impact of sampling variation and the paucity of mutant DNA fragments in blood and other fluids, full sequencing panels may be more broadly applicable to increase the number of detectable loci. That being said, comparisons of multi-target to single-target assays, to date, have not shown a difference in identifying molecular recurrence in colorectal cancer.^10^ Assay sensitivity is therefore likely to be more complex in clinical practice, especially when taking into account potential sites of disease-spread, such as the brain, where less ctDNA is found in the bloodstream.^11^

It will likely also be important to consider the context in which ctDNA is being used; for post curative-intent therapy and minimal residual disease (MRD) detection, sensitivity is critical to identify early disease recurrence. In the context of advanced disease, the level of ctDNA may be critical to inform early change to maximize the impact of available treatments. Similarly, some clinicians may continue treatment beyond progression in a selected group of patients on standard therapy if ctDNA levels are stable and/or reduced in an attempt to optimize immunotherapy outcomes.

Studies such as Eroglu et al.^1^ provide initial insights as to how ctDNA could be used in clinical practice to augment use of immunotherapy. In the adjuvant setting, baseline ctDNA profiling could be used as an integral selection biomarker to predict those patients who may derive the most benefit, mainly through enriching for patients at highest risk of relapse. A trial (NCT05060003) in stage II melanoma is currently investigating whether escalation to tiragolumab (anti-TIGIT) plus atezolizumab compared to atezolizumab could improve outcomes for patients with ctDNA-detected disease immediately following surgical resection. Further optimization of ctDNA assays may be needed to actualize this vision, especially given that a number of studies suggest that the percentage of patients with melanoma and detectable ctDNA immediately after surgery is low at approximately 15%.^12–15^ This is in contrast to some other tumor types, such as colorectal cancer, where ctDNA is more commonly identified in baseline post-surgery samples and strongly correlated with risk of relapse.^16^ This may be related to the site of initial metastatic spread because subcutaneous and nodal disease appears less likely to be associated with detectable ctDNA compared to visceral organs.^17^

Longitudinal use of ctDNA and aggregation of multiple biomarkers may be the most powerful ways to consider maximizing the potential of ctDNA. In melanoma, this might include inclusion of tumor mutational burden and interferon-γ gene expression signatures alongside ctDNA, because this composite biomarker has so far given the best performance characteristics in predicting adjuvant immunotherapy recurrence-free survival.^15^ In addition, longitudinal tracking of ctDNA may improve sensitivity.^13^ This approach is being tested in the DETECTION trial (NCT04901988; Table 1), which is examining whether a ctDNA-informed decision to administer investigator’s choice systemic therapy is noninferior compared with the standard of care clinical decision-making in the adjuvant setting for patients with stage IIB/IIC and III melanoma. This study conceptually aims to use ctDNA to reduce toxicity through sparing patients unnecessary adjuvant treatment while enriching for those with highest risk of relapse.

An alternative use of ctDNA in the adjuvant setting is to identify those patients who are not responding to adjuvant therapy or to detect molecular relapse following its completion. In the post-immunotherapy cohort (*n* = 10, stage II/IV) Eroglu et al.^1^ found that all of the ctDNA negative patients remained disease-free whereas the three patients with ctDNA detected had disease progression. The TebeMRD phase 2 trial (NCT05315258; Table 1) is currently investigating the safety and efficacy of treatment with tebentafusp in HLA-A*0201, DNA-detected disease (computed tomography scan negative) patients with uveal melanoma within 3 years of primary resection or cutaneous melanoma within 9 months of completion of adjuvant therapy.

In distant metastatic disease, a number of studies have shown that higher levels of baseline ctDNA are predictive of poor response to immunotherapy. This would suggest a possibility of developing intensification strategies that might be associated with increased toxicity but may be more likely to result in better cancer outcomes for patients with poor prognosis. Analysis of immune therapy response in larger cohorts of patients would be needed, with standardization across assays, to specify cutoffs defining poor prognosis. Composite biomarkers may be especially useful here including interferon-γ signatures.^18^ The CAcTUS trial (NCT03808441; Table 1) is examining an alternative approach in patients with *BRAF*^*V600*^ mutant melanoma and a variant allele frequency (VAF) of ≥1.5%. It hypothesizes that immune therapy responses could be maximized in tumors responding to targeted therapy. Patients are treated with targeted therapy until maximal response (≥80% drop in VAF) then switched to combination immune therapy.

An intriguing use of ctDNA may be through dynamic monitoring for early escalation of therapy in advanced disease. Eroglu et al.^1^ showed that patients with any increase in ctDNA level at weeks 3–11 on immunotherapy had a significantly shorter PFS, compared to patients with a decrease in ctDNA levels. Given that targeted therapy has been shown to be more effective when given at lower ctDNA levels, such monitoring could be very useful in BRAF mutant disease to facilitate switch from immunotherapy to targeted therapy.^4^ More data will be needed to assess whether other immunotherapy treatments such as tumor infiltrating lymphocytes could have greater benefit when given at molecular versus radiological relapse. Finally, in those patients who have responded to anti–PD-1–based therapy, ctDNA could be used to provide additional monitoring. This would provide further reassurance to those patients with ctDNA negative results^19^ and might raise the possibility of reducing the length of treatment. This would obviously have major economic advantages to the health system overall. Clinical trials are needed to better understand these questions, including whether retreatment with immune therapy or an alternative therapy would have better outcomes if treated when molecular progression is identified versus radiological progression.

There are a number of strategies that could potentially use ctDNA to maximize response to immune therapy and aid clinical decision-making regarding escalation or de-escalation of treatment. It is now possible to envisage, and in fact it appears likely, that the field is entering a new era where treatment decisions will be informed by dynamic assessment of tumor activity by peripheral blood monitoring.

## Figures and Tables

**Table 1:** Trials using ctDNA to inform treatment decisions in melanoma.

Trial name	Main hypothesis	Phase	NCT no.
Tiragolumab + atezolizumab vs. atezolizumab in the treatment of stage II melanoma patients who are ctDNA-positive following resection	Treatment with tiragolumab + atezolizumab in stage II patients with ctDNA-detected disease immediately following surgery have improved ctDNA clearance compared to atezolizumab alone.	II	NCT05060003
DETECTION	Treatment of patients with early-stage melanoma following surgery can be guided by longitudinal testing of ctDNA, which is noninferior to standard of care adjuvant therapy.	III	NCT04901988
TebeMRD	Patients with HLA-A*0201 and primary resected uveal melanoma or cutaneous melanoma following adjuvant therapy who have ctDNA detected will have molecular response to treatment with tebentafusp.	II	NCT05315258
CAcTUS	1. In BRAF mutant melanoma the efficacy of immune therapy is enhanced by response to pretreatment with MAPK pathway inhibition (dabrafenib plus trametinib).	II	NCT03808441
	2. Changes in ctDNA levels can be used to accurately inform when to switch from targeted to immune therapy.		

Abbreviations: CAcTUS, circulating tumor DNA-guided switch; ctDNA, circulating tumor DNA; DETECTION, circulating tumor DNA guided therapy for stage IIB/IIC/IIIA melanoma after surgical resection; TebeMRD, a phase 2 non-randomized, open-label, multi-center study of the safety and efficacy of tebentafusp in melanoma with molecular relapsed disease.
